# Propionic Anhydride Modification of Cellulosic Kenaf Fibre Enhancement with Bionanocarbon in Nanobiocomposites

**DOI:** 10.3390/molecules26144248

**Published:** 2021-07-13

**Authors:** Samsul Rizal, E. M. Mistar, A. A. Oyekanmi, Abdul Khalil H.P.S., Tata Alfatah, N. G. Olaiya, C. K. Abdullah

**Affiliations:** 1 Department of Mechanical Engineering, Universitas Syiah Kuala, Banda Aceh 23111, Indonesia; 2 School of Industrial Technology, Universiti Sains Malaysia, Penang 11800, Malaysia; abdulkan2000@yahoo.com (A.A.O.); tataalfatah83@gmail.com (T.A.); ck_abdullah@usm.my (C.K.A.); 3 Department of Industrial and Production Engineering, Federal University of Technology, Akure PMB 704, Nigeria; ngolaiya@futa.edu.ng

**Keywords:** propionic anhydride, kenaf fibre, bionanocarbon, characterisation, bionanocomposite

## Abstract

The use of chemical modification of cellulosic fibre is applied in order to increase the hydrophobicity, hence improving the compatibility between the fibre and matrix bonding. In this study, the effect of propionic anhydride modification of kenaf fibre was investigated to determine the role of bionanocarbon from oil palm shell agricultural wastes in the improvement of the functional properties of bionanocomposites. The vinyl esters reinforced with unmodified and propionic anhydride modified kenaf fibres bio nanocomposites were prepared using 0, 1, 3, 5 wt% of bio-nanocarbon. Characterisation of the fabricated bionanocomposite was carried out using FESEM, TEM, FT-IR and TGA to investigate the morphological analysis, surface properties, functional and thermal analyses, respectively. Mechanical performance of bionanocomposites was evaluated according to standard methods. The chemical modification of cellulosic fibre with the incorporation of bionanocarbon in the matrix exhibited high enhancement of the tensile, flexural, and impact strengths, for approximately 63.91%, 49.61% and 54.82%, respectively. The morphological, structural and functional analyses revealed that better compatibility of the modified fibre–matrix interaction was achieved at 3% bionanocarbon loading, which indicated improved properties of the bionanocomposite. The nanocomposites exhibited high degradation temperature which signified good thermal stability properties. The improved properties of the bionanocomposite were attributed to the effect of the surface modification and bionanocarbon enhancement of the fibre–matrix networks.

## 1. Introduction

Kenaf fibre is one of the most utilised natural fibres for non-woven products due to its intrinsic functional properties which include effective tensile and thermal properties [1]. Recently, kenaf fibre has been utilised as ideal choice material for the reinforcement of composites as an alternative to synthetic fibres [2]. The inherent biodegradability, renewability and non-toxicity potentials of kenaf fibres is attracting increasing attention. The utilisation of kenaf fibre as reinforcement agent in composite applications has been extensively reported in literature [3,4,5]. Ariawan et al. [6] incorporated kenaf fibre for the reinforcement of composites and reported improved mechanical and thermal properties compared to unreinforced kenaf fibre. The degradability properties of kenaf/epoxy composites fibre were investigated by Azwa et al. [7]. It was revealed that the addition of kenaf in the epoxy improved the thermal stability of the composite. Bernard et al. [8] investigated the effect of processing parameters on the mechanical properties of kenaf fibre incorporated in composite. It was revealed that the tensile properties of the composite were enhanced by 10% after the incorporation of kenaf fibre in the matrix. Thus, kenaf fibre has been used for reinforcements in composite applications [9] The efficiency and functional properties of reinforced composite depends on the interfacial interaction of fibre matrix in the composite formulation [10]. However, the major drawback in the reinforcement of composite using kenaf fibre is its inherent moisture absorption properties. Furthermore, the poor compatibility of fibre and hydrophobic matrix interaction resulting to low mechanical strength and thermal stability remains a challenge for researchers. To enhance functional properties of composites, surface modification of kenaf fibre is required for improved cell wall structures and cell wall chemistry [11]. In addition, the incorporation of nanofiller can improve the functional properties of nanocomposite [12]. Carbon-based composite provides beneficial functional properties due to its low density, high strength, and good thermal stability [13]. Li et al. [14] and Abdullah et al. [15] have reported that the use of nanoscale biomass in improvement of bionanocomposite compatibility has led to increased attention from researchers. Swaminathan et al. [16] and Galicia et al. [17] have revealed the high potential of nanocarbon materials in improvement of the mechanical properties of bionanocomposite. On the other hand, the incorporation of natural fibres in the carbon-based composite significantly enhances the functional properties of composite [18]. However, the impurities and non-polar compounds of natural fibre hinders the compatibility of composite which could reduce the interfacial fibre–matrix bonding. Chemical modification of natural fibre has the prospect to solve the challenge. Moreover, no study has been reported on the chemically modified propionic anhydride kenaf fibre in vinyl ester composite and enhancement of functional properties of bionanocomposite using nanocarbon from oil palm shell (OPS) as nanofiller. In the present study, nanocarbon from OPS was used to enhance the functional properties including the compatibility of fibre–matrix interaction and improved mechanical-thermal properties of the nanocomposite. The study aimed to fabricate and characterise nanocomposite prepared by incorporating modified propionic anhydride kenaf fibre in vinyl ester matrix and the enhancement of the functional properties by the addition of nanocarbon at different filler loading. Characterisation of the fabricated nanocomposite investigates the functional properties including the physical, mechanical, morphological, structural, and thermal properties to determine the influence of the nano filler in the fibre–matrix interaction of the bionanocomposite. 

## 2. Materials and Method

### 2.1. Materials

Nonwoven kenaf fibre mat with a thickness of 10 mm was used in the present study. The kenaf fibre was procured from Lembaga Kenaf dan Tembakau Negara, Kota Bharu, Kelantan, Malaysia. The OPS chips were utilised as the bio-nanocarbon precursor and were collected from Ulu Keratong palm oil mill, Segamat, Johor, Malaysia. Commercial vinyl ester comprising of 42% styrene monomer content, methyl ethyl ketone peroxide (MEKP) and cobalt naphthenate were supplied by Zarm Scientific & Supplies Sdn. Bhd., Malaysia. Propionic anhydride (C_6_H_10_O_3_), toluene (C_7_H_8_), ethanol (C_2_H_5_OH), acetone (C_3_H_6_O), potassium hydroxide (KOH) and hydrochloric acid (HCl) were obtained from Sigma Aldrich Subang Jaya, Selangor, Malaysia. All chemicals used in this study were of analytical grade.

### 2.2. Chemical Modification of Nonwoven Kenaf Fibre

**Figure 1:**
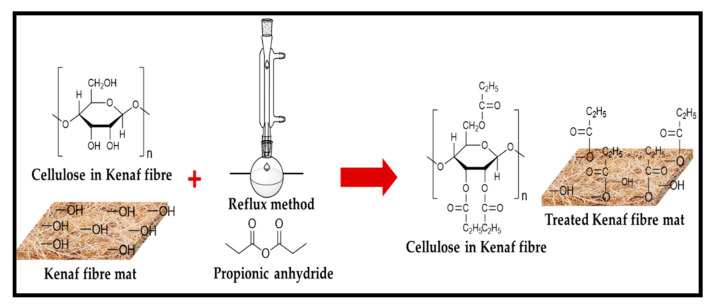
Schematic of chemically modified kenaf fibre.

The non-woven kenaf fibre mats of cross sectional dimension (200 mm × 200 mm × 5.5 mm) were used in the study. The fibres mats were placed in a soxhlet extractor and solvent extraction was achieved using toluene: ethanol: acetone. Ratio of 4:1:1 (*v/v*) within 3 h to obtain extractive-free fibres was adopted. The samples were dried in an oven for 24 h at 110 °C. Prior to weighing, samples were transferred to vacuum desiccator for 2 h and were allowed to cool to ambient temperature over silica gel. Five sets of the kenaf fibre mat were added to a flask containing a solution of the 100% propionic anhydride without dilution at three temperature reactions intervals of 80, 100, and 120 °C. Five different retention times intervals were set at 30, 60, 120, 180, and 240 min. The temperature reaction and retention time intervals were used according to previous studies on chemical modification process of biomass materials [19,20]. At the end of the reaction period, the flasks were removed from the oil bath, the hot reagent was decanted and the samples were washed using dry ethanol for another 3 h to get rid of remaining acid on the surface. The samples were air-dried for 2 h before oven drying for 24 h at 80 °C after which the weight was measured and recorded. The reaction outline is illustrated in Figure 1.

### 2.3. Preparation of Bionanocarbon

In this study, bionanocarbon with average particle size of 81.4 nm was obtained by chemical activation from oil palm agricultural waste which was generated from oil palm mill of oil palm shell (OPS). The precursor was washed with deionised water and then air-dried for 12 h to decrease the moisture to about 11%. The dried chips were crushed using a grinder equipped with a filter hole of 1.0 mm. The obtained particles were further ground using a Retsch mill with a filter of 0.25 mm. The grounded particles were sieved using 25 µm sieve size and were oven-dried at 110 °C for 24 h to eliminate inner moisture content before activation. The raw material was then mixed in KOH solution using an impregnation ratio of 1:0.5 (mass of raw materials to KOH mass). The mixture was then oven-dried at 120 °C for 4 h, the process was followed by carbonisation at 700 °C for 30 min in a muffle furnace. The bio nanocarbon obtained was cooled to ambient temperature in a desiccator and then washed with 0.1 M HCl solution at 85 °C. After which it was rewashed with hot distilled water until pH∼7 was achieved. Finally, the yielded bio nanocarbon was dried at 110 °C for 24 h. Nanostructured nanocarbon was produced using high-energy ball milling (horizontal ball milling) at a rotational speed of 170 rpm for 24 h in an ambient environment. The stainless-steel chamber was loaded with a ratio of ball to carbon powder of 10:1 (*w/w*). The balls were made of stainless steel with 20 mm, 12 mm, and 10 mm diameter. After then, the bionanocarbon was oven-dried at 110 °C for 24 h and kept in a glass vial inside the desiccator. 

### 2.4. Characterisation of Bio Nanocarbon and Kenaf Fibre

Characterisation studies were carried out to investigate the functional properties of the chemically modified kenaf fibre including the prepared bionanocarbon and fabricated nanocomposites. The absorption of propionic anhydride onto nonwoven kenaf fibre mats were determined from the weight percent gain (WPG) by the samples after the modification. Weight gain was determined from the percentage of oven dried weight of the chemically modified fibres according to Equation (1). The contact angle of unmodified and modified nonwoven kenaf fibres were measured using the sessile drop method on KSC CAM 101 (KSV Instruments Ltd., Espoo, Finland) at room temperature. The morphologies of the nonwoven kenaf fibres were analysed using FESEM (FEI Quanta FEG 650, Thermo Fisher Scientific, Eindhoven, The Netherlands). The structural analyses of nonwoven kenaf fibres and nanocarbon were obtained using an FT-IR Prestige-21 spectrophotometer (Shimadzu, Chiyoda-ku, Tokyo, Japan). The thermogravimetric analysis (TGA) of the nonwoven kenaf fibres and bio nanocarbon were performed using Mettler-Toledo thermogravimetric analyser model TGA/DSC 1 (Mettler Toledo, Schwarzenbach, Switzerland). The textural composition of bionanocarbon and morphological properties were obtained using the transmission electron microscopy (TEM), which was investigated using an energy-filtered EFTEM Libra 120—Carl Zeiss instrument (Oberkochen, Germany). The surface physical properties of bio nanocarbon was characterised with Micromeritics ASAP 2020 analyser (Norcross, GA, USA) using N_2_ as the adsorbate at 77 K according to the Brunauer-Emmet-Teller (BET) method.

(1) $$\mathrm{Weight}\;\mathrm{percent}\;\mathrm{gain}\;(\%)=\frac{\mathrm{Weight}\;\mathrm{gain}}{\mathrm{Original}\;\mathrm{weight}}\;\times \;100$$

### 2.5. Preparation of Nanocomposites

The modified nonwoven kenaf fibre with the temperature reaction at 100 °C and retention time at 180 min was used as component mixture in the fabrication of nanocomposites at the optimum WPG. The composites of vinyl ester reinforced with unmodified or modified kenaf fibres were incorporated using 0, 1, 3, 5 wt% of bio-nanocarbon (parts per hundred resin) fixed at 40% of kenaf fibres loading [21]. About 0.2 wt% of cobalt naphthenate and 1.5% wt% MEKP were used as an accelerator and as hardener/catalyst, respectively [22]. The nanocomposites reinforced with unmodified and modified nonwoven kenaf fibres were filled with 0, 1, 3, and 5 wt% of activated bio-nanocarbon and were labelled as VE/UK/NC0, VE/UK/NC1, VE/UK/NC3, VE/UK/NC5, VE/MK/NC0, VE/MK/NC1, VE/MK/NC3, VE/MK/NC5, respectively. The composites were fabricated by compounding the matrices with nanofillers and homogenised using a mechanical stirrer. The nanocomposites were fabricated via the resin transfer moulding (RTM) technique. The kenaf fibres were then placed inside the mould cavity of dimension 200 mm × 200 mm × 5.5 mm. The samples were moulded with a Hypaject Mark II RTM injection system (Plastech, Thermoset Tectonics, Gunnislake, UK). The nanocomposites were moulded at room temperature at the beginning of resin injection with 200 kPa vacuum pressure. The obtained nanocomposites were cured at room temperature for 24 h and post-cured at 80 °C in an oven for 4 h. The compressed composites were cooled to ambient temperature in a desiccator containing granulated silica gel. Afterwards, they were moulded to test samples and were placed in a zip lock bag before storing in a desiccator for further analysis.

### 2.6. Characterisation of Bio-Nanocomposites

The characterisation was conducted on the fabricated bio nanocomposite to investigate the functional properties. The physical properties were obtained using density profile and wettability tests. The density profile of the specimens with the thickness of bio-nanocarbon was investigated using X-ray density Profiler Grecon model DA-X (Alfeld, Germany). The bio-nanocarbon composite samples with a 50 mm × 50 mm × 5.5 mm cross-sectional dimension were cut in a chamber at room temperature. The relative humidity before the investigation was measured at 30 ± 2%. During scanning, the specimen was inserted into the cassette holder of each batch scan and analysed at 0.025 mm/s. 

The water absorption and swelling thickness analyses of the test samples were investigated according to standard methods. The water absorption test was conducted to investigate the water intake capacity of the bio nanocomposite. The thickness swelling test was conducted to evaluate the swelling of the samples. The tests were carried out according to ASTM D570 for all composite test samples [23]. Before the investigation was carried out, the weight and thickness of each specimen were recorded. Five test samples of bio-nanocomposite were immersed in distilled water at ambient atmosphere for 24 h. The samples were then taken out and filter papers were used to remove the excess water on the surface before the weight and thickness were recorded. The rate of water absorption percentage was determined from Equation (2), and the percentage thickness swelling was calculated according to Equation (3).

(2) $$\mathrm{Water}\;\mathrm{absorption}\;(\%)=\frac{W_{2}-W_{1}}{W_{1}}\;\times \;100$$

where *W*_1_ is the weight of the samples before immersion and *W*_2_ is the weight of samples after immersion.

(3) $$\mathrm{Water}\;\mathrm{absorption}\;(\%)=\frac{W_{2}-W_{1}}{W_{1}}\;\times \;100$$

(4) $$\mathrm{Water}\;\mathrm{absorption}\;(\%)=\frac{W_{2}-W_{1}}{W_{1}}\;\times \;100$$

(5) $$\mathrm{Thickness}\;\mathrm{swelling}\;(\%)=\frac{T_{2}-T_{1}}{T_{1}}\;\times \;100$$

where *T*_1_ and *T*_2_ are the thickness of the samples before and after soaking. 

The mechanical properties of the bio nanocomposites were analysed in terms of tensile, flexural, and impact tests. The tensile strength, tensile modulus, and elongation at the breaking point of the prepared bio nanocomposites were measured according to ASTM D638 specifications [24]. The flexural strength and modulus properties were investigated according to ASTM D790 standard [25]. The tensile and flexural tests were conducted using an INSTRON 5582 universal testing machine (Norwood, MA, USA). The impact strength was conducted using ASTM D256 for the Izod test [26]. The samples were notched using V-notch type with depth of 2 mm and radius of 0.25 mm at the centre before they were tested on a Gotech testing machine, Model GT-7045 MD (Taichung City, Taiwan). The five test specimens were evaluated, and the average values were recorded for each sample composition. 

The morphological study of tensile fracture sample was observed using FESEM (FEI Quanta FEG 650, Thermo Fisher Scientific, Eindhoven, The Netherlands). A thin section of the samples was mounted on an aluminium (Al) stub holder with double-sided copper (Cu) tape holder. The films were coated with a platinum (Pt) layer using sputter and coater Quorum Technologies Q150T to enhance their electrical conductivity. The FESEM micrographs were examined at an accelerating voltage of 10 kV under conventional secondary electron imaging stipulations.

The functional group analysis of the bio nanocomposites was investigated using the Fourier transformation infrared (FT-IR). The analysis of test samples was obtained using FT-IR Prestige-21 spectrophotometer (Shimadzu, Chiyoda-ku, Tokyo, Japan). The composite powder samples were prepared and oven-dried at 60 °C for 24 h before the analysis was conducted. The powder was mixed with KBR and was pressed into the circular film before it was placed in the FT-IR machine and the transmittance was obtained. The spectra were measured at the range of wavenumber from 4000–400 cm^−1^ and at a resolution of 4 cm^−1^. 

The thermal properties of the bio nanocomposites were examined using Mettler-Toledo thermogravimetric analyser model TGA (Mettler Toledo, Schwarzenbach, Switzerland) for the TGA. About 10 mg of bionanocomposite was weighed in an alumina crucible and was put in a thermogravimetric analyser with a pre-weighed empty alumina crucible as a reference. The thermal analysis was conducted within furnace temperature range of 30 to 800 °C, at heating rate of 10 °C/min under the flow nitrogen (N_2_) at a 50 mL/min flow rate. The results obtained were interpreted and were derived through the STAR^e^ SW 10.00 software program for the evaluation of the onset temperature (T_on_) and maximum temperature (T_max_) of decomposition, including the percent mass loss (%)

## 3. Results and Discussion

### 3.1. Characterisation of Nonwoven Kenaf Fibre

#### 3.1.1. Weight Percent Gain Properties

**Figure 2:**
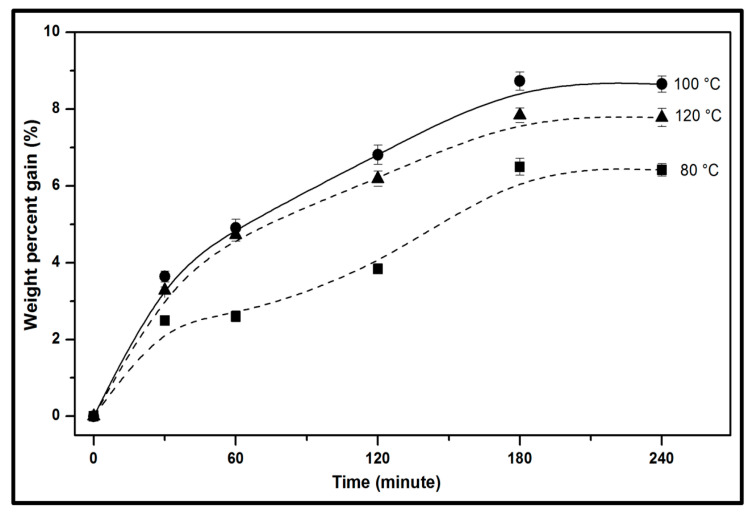
Weight percent gain of modified nonwoven kenaf fibre with different reaction temperature and different retention time.

The weight percent gain (WPG) of kenaf fibre after the chemical modification as a function of time at different interval of temperature is illustrated in Figure 2. In general, the non-woven kenaf fibre exhibited considerable weight gain as time was increased at the initial stage until saturation was reached irrespective of the difference in temperature, indicating that the swelling of the fibre may inhibit interfacial bonding and may likely result to cracking at the adjacent matrix beyond 180 min [6]. In the reaction profile, increase in WPG was achieved at the initial stage due to relative reaction of hydroxyl groups and the rate diffusion of reagents in the fibre–matrix interface [27]. As can be seen, WPG increased as the reaction time and reaction temperature increased. However, further increase in the reaction temperature at 120 °C indicated a decrease in WPG [7]. As the reaction time was increased from 60 min to 180 min, spontaneous weight gain was achieved beyond which steep decrease in WPG was exhibited by the kenaf fibre. The decline in WPG due to the effect of increased reaction temperature could be as a result of the effect of degradation of the cellulose, hemicelullose and lignin composition of the kenaf fibre [28]. Therefore, to minimise the possibility of fibre degradation, the optimum reaction temperature of kenaf fibres should not be exceedingly high and should sufficiently permit rapid reaction. A reaction temperature at 100 °C provided an optimum weight gain as exhibited by the kenaf fibre. 

The initial phase from 60 min until 180 min indicated a high rate of reaction due to the availability of a higher number of hydroxyl (-OH) groups during the initial reaction time. At 180 min of reaction time, it was revealed that the propionic anhydride modified the diffusion into the cell wall due to effect of the rate of reaction time and temperature. The increase in WPG could be attributed to a balance of moisture loss and the addition of active groups of the propionic anhydride which increased as the reaction time increased reaction rate. It could be deduced that at the beginning of the reaction time, the -OH groups were preferentially replaced. As the reaction time increased, the propionic anhydride dissolved in the interface, the swelling ability as a result of the increased reaction time influenced the diffusion into the unreacted -OH groups on the surface of the fibre [29]. The effectiveness of treatment increased in the form of weight percent gain until 180 min, and after which it remained constant. The samples from optimum retention time indicated that the optimum retention was achieved at 3 h of reaction at 100 °C, which indicated the reaction period beyond 3 h had no significant effect of the WPG exhibited by the propionic modified kenaf fibre. 

#### 3.1.2. Morphological Analysis

**Figure 3:**
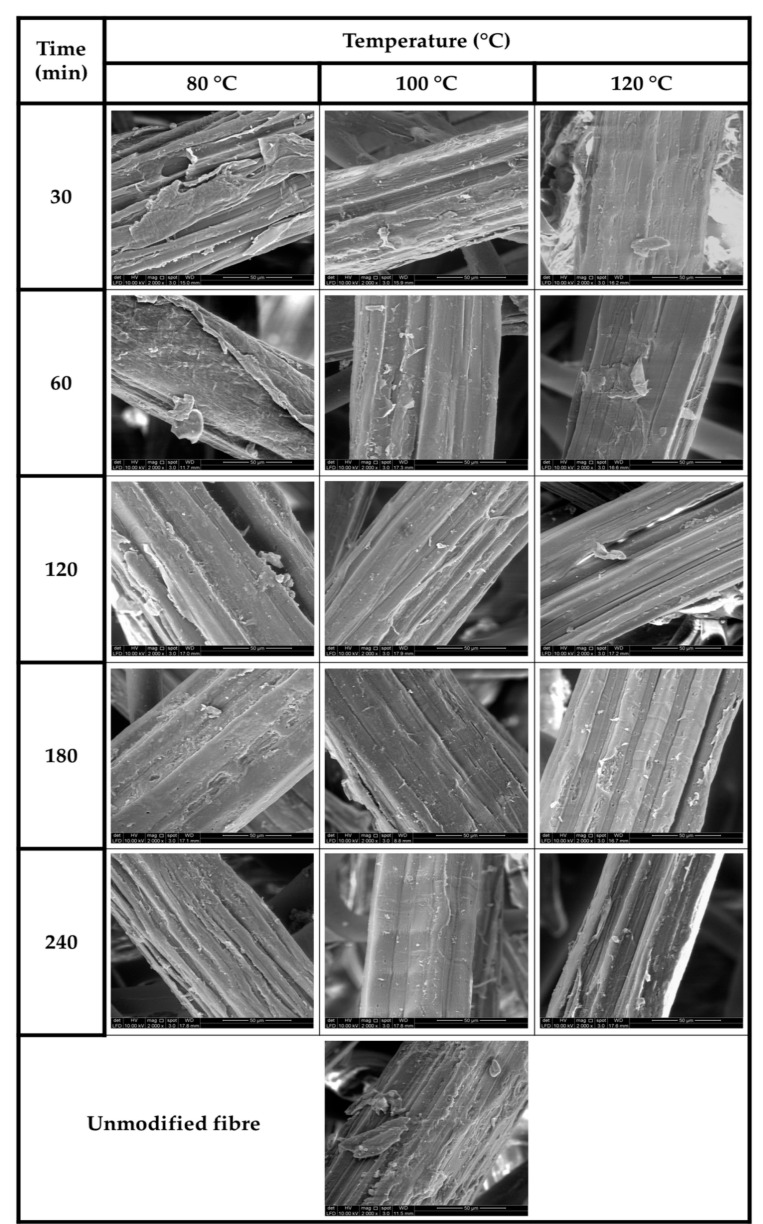
SEM micrographs of modified nonwoven kenaf fibre with different reaction temperature and different retention time.

The morphological surface properties of the modified kenaf fibre at different reaction periods are illustrated in Figure 3. At the same retention time, increase in reaction temperature exhibited by the kenaf fibre increased the textural surface properties of the fibre. It was revealed that as the reaction temperature was increased at 30 min reaction period, the presence of wax, oil and other forms of impurities decreased as the temperature increased. The impurities inhibited fibre–matrix bonding interaction. An improved surface as the reaction temperature was increased from 80 to 100 °C indicated better interaction at the interfacial region of the reinforcing fibres [30]. The reaction temperature at 100 °C indicated a smoother and cleaner surface compared to morphology of fibre at 80 °C, this could be attributed to the removal of surface impurities affecting reactive chemical groups in the interface. However, as the reaction time a reaction temperature was increased, the surface morphology of propionylated modified kenaf fibres indicated reduction of impurities such as silica, non-cellulosic materials, inorganic substances and improved interfacial bonding within the fibre–matrix structure. This resulted in improved void spaces, which could promote the functional properties of the fibre as reinforcement material [31]. The effect of the modified kenaf fibre surface and the removal of surface impurities provided significant change in morphology of fibre surfaces and enhanced the fibre–matrix adhesion. 

#### 3.1.3. Structural, Wettability, and Thermal Properties

**Figure 4:**
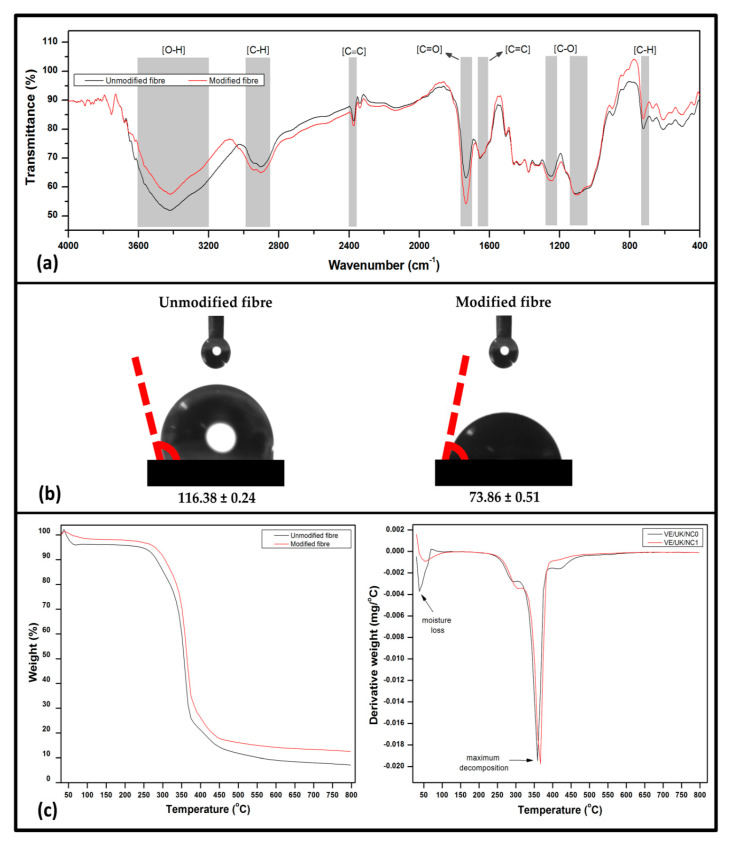
Characterisation of unmodified and modified nonwoven kenaf fibre (**a**) FT-IR, (**b**) contact angle, and (**c**) thermal analysis.

The functional properties, wettability and thermal properties of the modified and unmodified kenaf fibre are illustrated in Figure 4. The chemical structure of the modified and unmodified kenaf fibre was analysed using FT-IR spectra (Figure 4a). A shift in the peaks denoting the functional groups exhibited by the kenaf fibre was as a result of the surface modification. A strong peak was indicated around 3400–3800 cm^−1^ for both modified and unmodified kenaf fibre indicating the presence of O-H stretching vibration [32]. However, the increased intensity of the peak around 3750 cm^−1^ signified increased hydroxyl groups in the fibre structure as a result of the effect of the modification by propionic anhydride [33]. This was demonstrated from the wettability properties of the modified kenaf fibre compared to unmodified kenaf fibre in Figure 4b. A broader peak for the modified kenaf fibre signified the presence of C=C and C=O stretching vibrations around 2400 cm^−1^ and 1750 cm^−1^, respectively. The peak around 1500 cm^−1^ illustrated the presence of lignin in the kenaf fibre [34]. A small peak at 1250 cm^−1^ was assigned to C-O stretching vibration in the modified kenaf fibre which indicated that lignin was partially removed from the fibre surface [35]. The effect of propionic anhydride modification provided change in chemical structure of the fibre as revealed especially by the presence of C=O and C-H groups. The peaks at 850 cm^−1^ in the unmodified kenaf fibre was assigned to CH out-of-plane vibrations in substituted ethylenic system.

As indicated in Figure 4b, the wettability properties of the modified and unmodified kenaf fibre were illustrated from the contact angle analysis. It was revealed that the unmodified kenaf fibre exhibited higher contact angle compared to the modified kenaf fibre. It could be deduced that the unmodified kenaf fibre exhibited lower wettability due to the hydrophobic substances such as wax and lignin [36]. This is attributed to the non-polarity of the lignin and wax component in the fibre component. However, the surface modification of the fibre by propionic anhydride resulted in the removal of the non-polar components of the kenaf fibre thereby leading to increase in the polarity of the fibre structure. Therefore the effect of the modification significantly enhanced dispersion due to increase in surface energy influencing fibre–matrix bonding interaction [37]. Furthermore, chemically modified fibres had increased wettability properties compared to unmodified fibres due to better compatibility between the fibre and the matrix as a result of less voids. In addition, the wettability of the surface also depends on the reactivity of the fibre surfaces. During propionylation, the attachment of carboxyl groups and dispersive aliphatic chain (–CH_2_–CH_3_) were incorporated at the surface. This functional group has the possibility of indirectly affecting the wetting properties between the fibres and the surface. 

The thermal properties of the unmodified and modified kenaf fibre are illustrated using temperature profile from TGA analysis (Figure 4c). The initial weight loss attributed to evaporation was achieved within 100 °C for both unmodified and modified kenaf fibre. At this stage, small variation in the percent weight loss was achieved. The water loss was associated with the fibre humidity even though the hydrophilic properties of the fibre-inhibited total water loss due to structurally bound molecules within the fibre network [38]. The first stage of decomposition was achieved between endothermic 250–300 °C. A noticeable decrease in weight loss was observed in the unmodified kenaf fibre relative to the modified fibre. The weight loss as exhibited by the modified fibre could be attributed to the effect of propionic anhydride modification which decreased the hydrophilicity of the fibre. As a result, the fibre loss was indicated in the DTG curve. The modification of the fibre provided interface for the interaction of anhydride groups and cellulosic OH groups [39]. The second stage of decomposition was achieved within 370 and 400 °C. At this stage, maximum weight loss was achieved due to the volatilisation of filler reinforcement kenaf fibre as indicated in the DTG curve for both modified and unmodified fibre [40]. In general, there was a noticeable shift to a higher temperature between unmodified and modified fibre, respectively, as it is indicated in the decomposition temperature and maximum temperature of degradation of the fibre. The Tonset shifted from 336.32 °C (unmodified) to 352.35 °C (modified) and maximum decomposition temperature shifted from 349.72 °C to 365.75 °C, respectively. This suggested that the modified kenaf fibre could improve on the thermal stability of the structure of the nanocomposite since it can be deduced that thermal stability of a material could be enhanced at higher temperature. The higher decomposition temperature possibly was attributed to the interfacial interaction between the anhydride groups and the hydrophilic groups on the fibre surface [41].

### 3.2. Characterisation of Bio Nanocarbon

**Figure 5:**
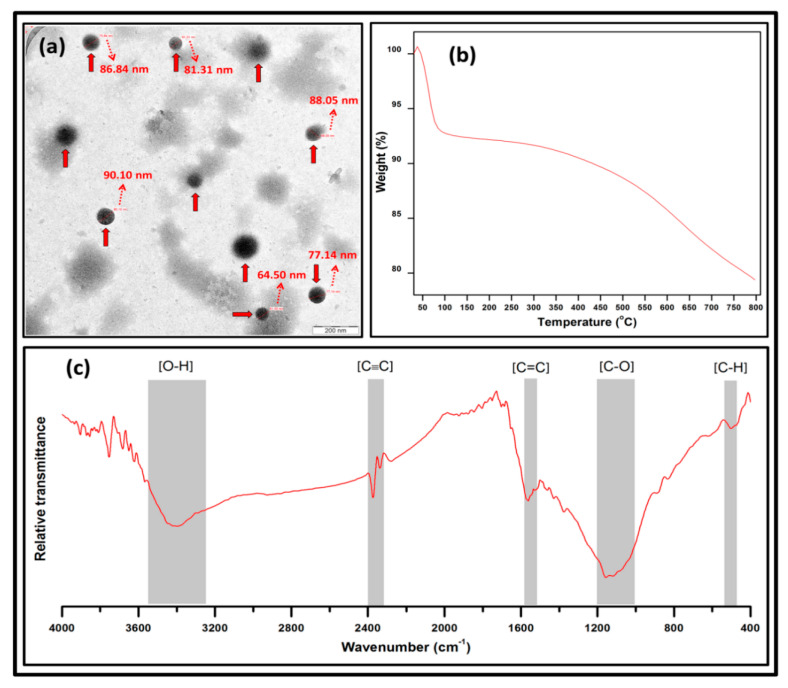
Characterisation of bio nanocarbon (**a**) TEM, (**b**) TGA, and (**c**) FT-IR analysis.

The morphological, thermal and functional properties of the synthesised bio nanocarbon were analysed from the TEM, TGA and FT-IR studies, respectively. The morphological features of the bio nanocarbon indicated uniformly distributed particle sizes ranging from 64.50 nm to 90.10 nm in Figure 5a; similarly, there were black colouration patches across the boundary observed. The micrograph of the bionanocarbon nanofillers exhibited good level of dispersion. It is highly likely that the uniformly distributed particle sizes of the nanocarbon could enhance the compatibility between the fibre–matrix interactions and also could potentially fill the voids, cracks within the interfacial interaction, resulting in enhanced functional properties of the bio nanocomposite [42]. The percent weight loss of nanocarbon as a function of temperature is indicated in Figure 5b. The TGA curve illustrated an extremely slight mass loss of nanocarbon. The first weight loss was within 100–150 °C which was attributed to the removal of free hydrogen bonded water molecules [43]. The initial weight loss was followed by major weight loss at initial decomposition temperature of 572.83 °C. At this stage upon decomposition, the oxygen functional groups on the surface of the nanocarbon was converted to oxides of carbon and was liberated from the nanocarbon in gaseous form [44]. The removal of hydroxyl groups and secondary reaction with adjacent carboxyl groups occurred upon heating. A total yield of 79.39% was obtained at 800 °C. The functional properties of the nanocarbon were obtained in transmittance mode (Figure 5c). The FT-IR spectra of the nanocarbon around 2400 cm^−1^ revealed the presence of C=O assigned to carbonyl groups. The spectra around 1600 cm^−1^ was assigned to the C=C stretching vibration. A small peak at 1175 cm^−1^ correspond to C-O-C stretching vibrations in esters [45]. The characteristic peaks between 3550 and 3610 cm^−1^ indicated the addition of some oxygen functional groups to the fibre surface by the oxidation of unsaturated carbon atoms for improved compatibility of fibre–matrix interaction [46]. 

### 3.3. Characterisation of Nanocomposites

Characterisation studies were conducted to investigate the functional properties of the synthesised propionic modified kenaf/vinyl esters/nanocarbon enhanced nanocomposites. The physical, mechanical, surface morphological, chemical structure and thermal analysis of the nanocomposites were examined. 

#### 3.3.1. Physical Properties

**Figure 6:**
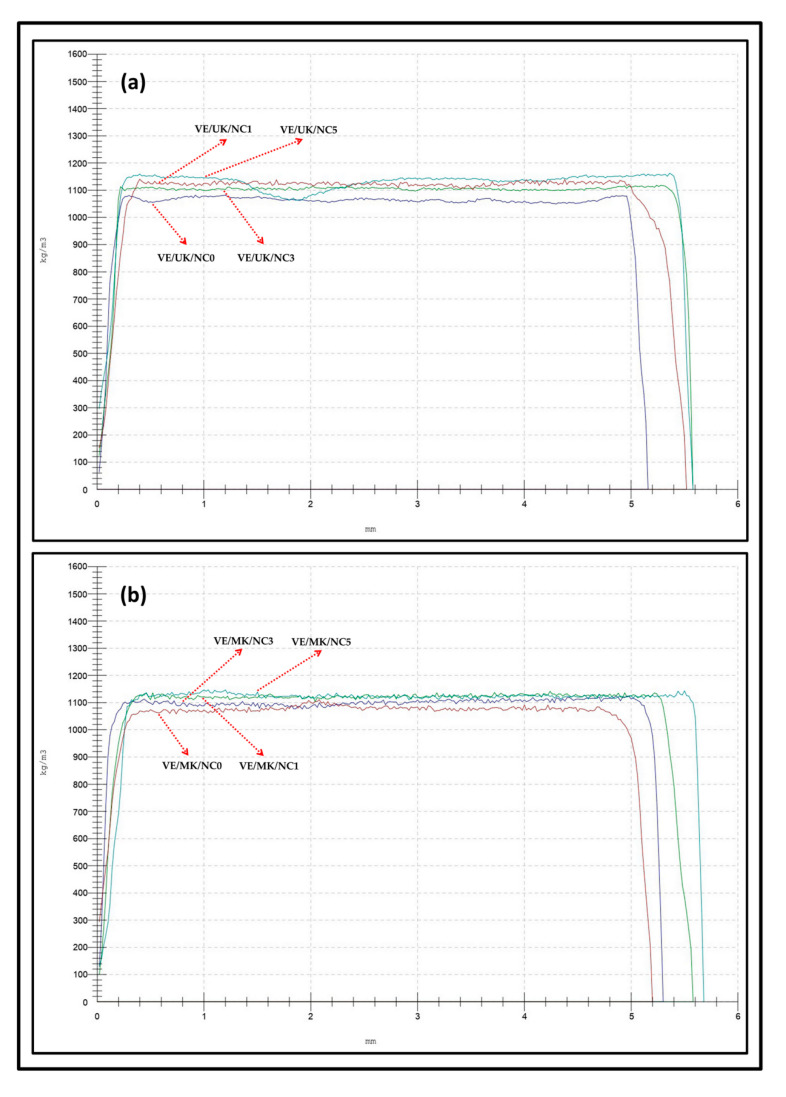
Density profile analysis of (**a**) unmodified and (**b**) modified kenaf reinforced nanocomposite.

The density profile analysis of the nanocomposite at different filler loading was compared to the controlled sample. It was revealed according to Figure 6 that the controlled unmodified kenaf-based nanocomposite exhibited a density of 1032.23 kg/m^3^ and increased as the filler loading increased. Optimum density of the unmodified kenaf derived nanocomposite was obtained at 5% filler loading with an equivalent density of 1101.89 kg/m^3^ (Figure 6a). It is generally noticed that the voids between the fibre–matrix interface was reduced with the addition of filler loading which implies that the nanofiller improved interfacial interaction of nanocomposite structure. However, the significance of the propionic derived kenaf nanocomposite in Figure 6b indicated a better compatibility between fibre and matrix network compared to the unmodified kenaf-based nanocomposite. This was exhibited in the increased density demonstrated by the modified nanocomposite. The compatibility was further increased as the filler loading increased as evident in the increased density. The voids between fibre–matrix decreased as the filler loading increased as a result of increased density. The optimum filler loading at 5% and at a density of 1089.63 kg/m^3^ enhanced better compatibility and improved interfacial interaction in the nanocomposite networks. Generally, the stability of peak altitude and width influenced higher recorded density value, indicating that modified fibre provided superior compatibility as a result of less void and better adhesion interaction between the fibre and the matrix. In addition, the bio-nanofillers were homogenously spread across the surface of the composite and occupied available sites, voids and pore networks within the filler-matrix interaction resulting to the formation of interlocks within the composite materials.

**Figure 7:**
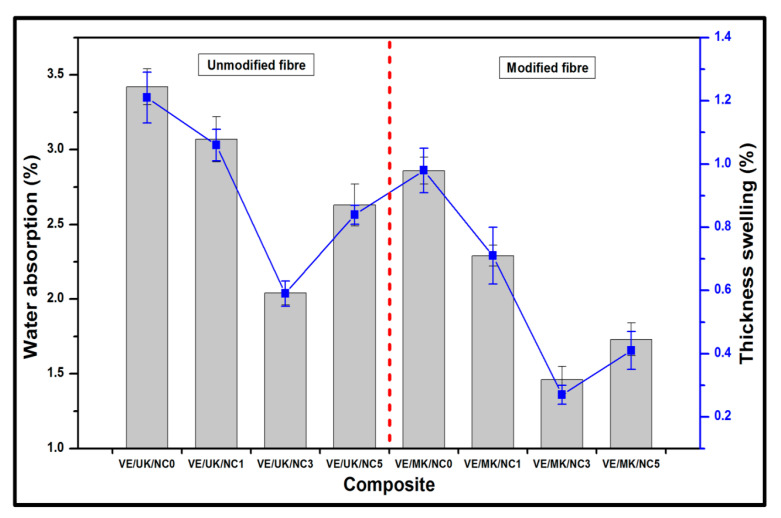
Water absorption and swelling properties of unmodified and modified kenaf reinforced nanocomposite.

The percentage water absorption and thickness swelling of the synthesised unmodified kenaf bio nanocomposites and modified kenaf fibre nanocomposite is indicated in Figure 7. It was indicated that the percent water absorption and swelling decreased as the filler loading increased compared to the control sample. The decrease observed was attributed to the addition of nanofiller in the fibre–matrix interaction within the nanocomposite networks [47]. It was indicated that the optimum decrease in the water absorption and swelling properties of the unmodified kenaf reinforced nanocomposite were achieved at 3% filler loading, which indicated that better compatibility of the fibre–matrix interaction as a result of the decrease in the voids in the interface was enhanced at this filler loading [48]. However, beyond 3% incorporated nanofiller, poor dispersion in the fibre–matrix networks was achieved which consequently resulted in increased water absorption and thickness swelling properties at 5% nanofiller loading. Comparatively, an observable trend of decreased water absorption and thickness swelling was achieved with the propionic anhydride modified kenaf reinforced nanocomposite. The effect of modification decreased the voids in the fibre–matrix interaction and resulted in a decrease in the hydroxyl groups in the interface suggesting improved fibre–matrix bonding. A similar trend indicating optimum decrease in water absorption and thickness swelling was also achieved at 3% nanofiller loading, even though at this loading condition a more reduced percent water absorption and thickness swelling was noticed in the propionic anhydride modified kenaf nanocomposite, which indicated better compatibility and enhanced reinforcing ability. 

#### 3.3.2. Mechanical Properties

The tensile properties, which include tensile strength, tensile modulus, elongation at break (EAB), tensile toughness, flexural strength, flexural modulus, flexural toughness and impact strength, were examined to determine the effeciveness of the mechanical properties of the unmodified kenaf-based nanocomposite and propionic anhydride modified derived nannocomposite as presented in Table 1. The average tensile strength and tensile moduls was revealed to increases with the incorporation of nanocarbon in the fibre–matrix interface in the unmodified kenaf-based nanocomposite compared to the controlled nanocomposite. The increase of bionanocarbon up to 3% loading ultimately resulted in the decrease in voids within the fibre–matrix interaction, hence, increase in the compatibility of the nanocomposite structure. The good compatibility improved the tensile strength and tensile modulus of the nanocomposites. The obtained results indicated higher tensile properties of the bio nanocomposites having modified fibre compared to unmodified fibre produced bionanocomposite. It can be concluded that chemical modifcation of the fibre improved the compatibility of the nanocomposites. Enhanced compatibility was achieved at 3% filler loading for the modified and unmodified kenaf reinforced nanocomposite with equivalent tensile strength and tensile modulus of 68.65 ± 0.79 and 78.61 ± 1.02 MPa and 2.65 ± 0.06 and 2.87 ± 0.07 GPa, respectively. Chemical modification of the fibre surface has been reported to increase the tensile strength of reinforced natural fibre-based nanocomposite [49]. Beyond 3% filler loading poor dispersion and interfacial interaction could be atrributed to the decreased tensile strength in both unmodified and modified kenaf reinforced nanocomposite. The enhanced tensile properties attributed to the effective mechanical interlocking between fibre and matrix, and dipolar interactions between anhydride groups and cellulosic hydroxyl (-OH) group [50]. Hence, with the good interfacial bond interaction, improved resistance to fibre pullout without rupturing the fibre enhanced the tensile properties. Similar trend was noticed in the tensile toughness, flexural strength, flexural modulus, flexural toughness and impact strength as the filler loading was increased indicating that enhanced compatibility of fibre–matrix structure was enhanced until 5% in the case of unmodified nanocomposite even though there was better compatibilty and enhanced mechanical properties at this filler loading as indicated in the propionic anhydride reinforced nanocomposite compared to the unmodified nanocomposite. Conversely, the EAB decreased as the filler loading increased in both unmodified and modified kenaf reinforced nanocomposite. The incorporation of 3% filler loading indicated optimum decrease in percent EAB for both unmodified and modified kenaf reinforced nanocomposite. Although, at this filler loading, the propionic anhydride nanocomposite exhibited less percent EAB. This signifies that improved compatibility of the fibre–matrix bonding interaction was achieved as the EAB decreased. In conclusion, it was revealed that the incorporation of the nanofiller in the fibre–matrix interface enhanced the mechanical properties of the bionanocomposites compared to the controlled bionanocomposite, i.e without activated bio-nanocarbon. A progressive increase in tensile toughness, flexural strength and flexural modulus as filler loading increased could be attributed to the effect of hydrogen bonding within the composite network between filler and matrix resulting to a cohesive chain which indicates that activated bionanocarbon in the filler-matrix interaction was strongly related to the surface functionality of the carboxyl and hydroxyl groups.

#### 3.3.3. Morphological Analysis

**Figure 8:**
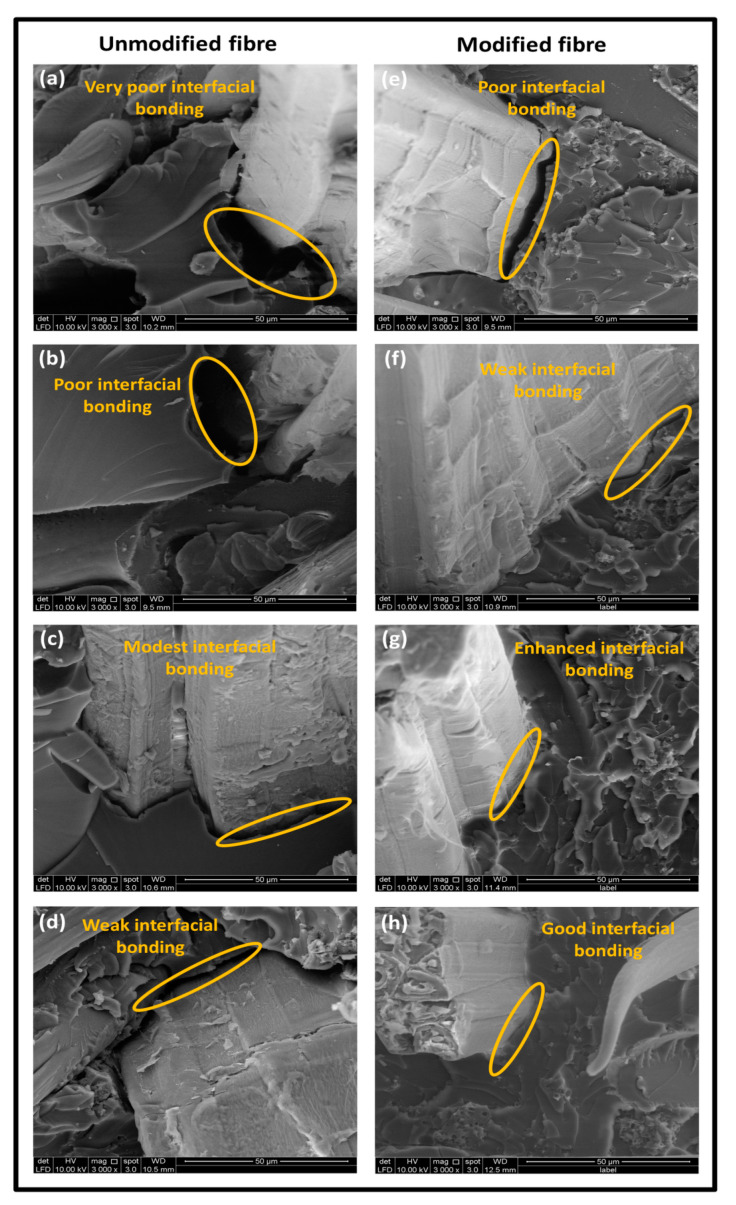
FESEM micrograph of tensile fracture sample of (**a**) VE/UK/NC0 (**b**) VE/UK/NC1 (**c**) VE/UK/NC3 (**d**) VE/UK/NC5 (**e**) VE/MK/NC0 (**f**) VE/MK/NC1 (**g**) VE/MK/NC3 and (**h**) VE/MK/NC5.

The morphological properties of the unmodified and modified kenaf reinforced nanocomposite are shown in Figure 8. As expected, the unmodified nanocomposite exhibited relatively poor interfacial fibre–matrix interaction as a result of the cracks and voids predominantly across the boundaries indicating fibre fracture and fibre pull out within the interface (Figure 8a,b). Unmodified composite exhibited noticeable fracture surfaces. This observation clearly indicated that unmodified fibre and vinyl ether matrix have poor adhesion and weak fibre–matrix bonding interaction. However, as the nanofiller loading was incorporated, improved fibre–matrix bonding interaction was achieved across the interface. 

**Figure 9:**
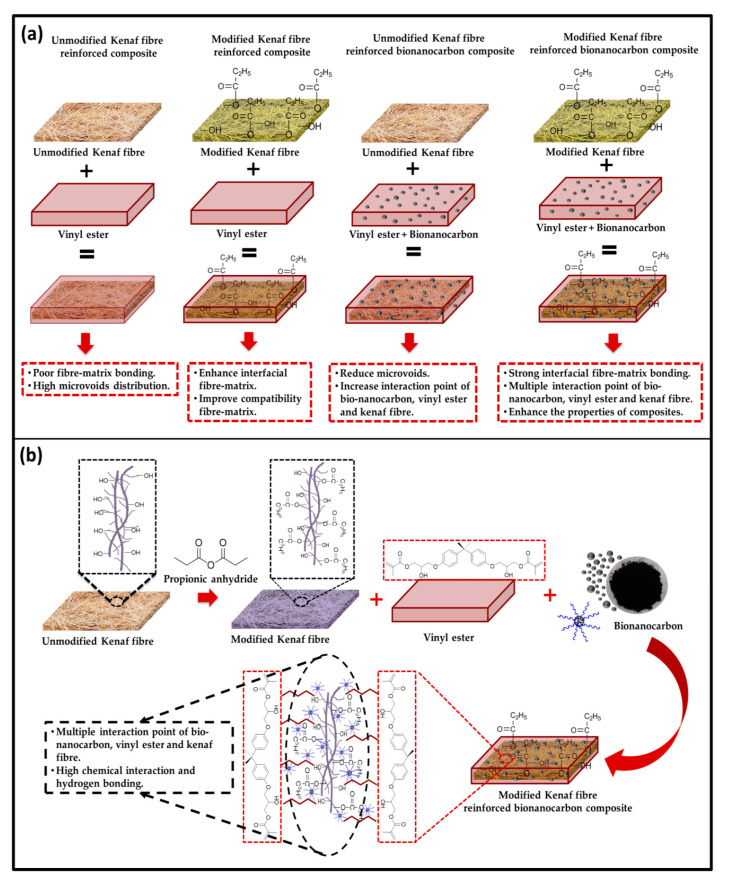
Possible Mechanism of bionanocarbon enhanced reinforced kenaf-based nanocomposites indicating (**a**) Unmodified and modified kenaf fibre reinforced composite with bionanocarbon (**b**) propionic anhydride modified Kenaf fibre reinforced composite with bionanocarbon.

As the nanofiller was increased by 3%, improved compatibility due to the well embedded fibres by the addition of bio nanocarbon in the available voids within fibre–matrix interface. This created an enhancement in the interfacial fibre–matrix bonding as observed in Figure 8c. On the other hand, there was weak interfacial bonding as a result of fibre pull out in the matrix network as evident in Figure 8d. The effect of propionic anhydride modification of the kenaf in the matrix networks created a poor interfacial bonding without nanocarbon (Figure 8e). The improvement of fibre–matrix bonding interaction increased as the filler loading increased from 1%, 3% and 5% respectively as can be seen in (Figure 8f–g). However, good compatibility of the interfacial interaction of fibre and matrix network was achieved as the nanofiller loading was increased by 5% [32]. In Figure 9, the unmodified kenaf fibre in vinyl ester matrix exhibited poor interfacial bonding due to voids distributed predominantly across interfacial boundaries. Naturally, fibres consist of embedded layers such as waxy substances. It was clear from the possible mechanism that decrease in microvoids as a result of the incorporation of bionanocarbon in the unmodified kenaf fibre in the vinyl ester matrix improved the compatibility and properties of the nanocomposite. Furthermore, chemical modification, dissolution of waxy substances improved the interaction of the –OH and the –COOH groups on the fibre surface which resulted in increased polar–polar interaction within the matrix. In addition, the strong interface between fibre and matrix has the tendency of inhibiting interfacial slippage due to the enhancement of the fibre–matrix bonding interaction as a result of the incorporation of bionanocarbon.

#### 3.3.4. Chemical Structural Analysis

**Figure 10:**
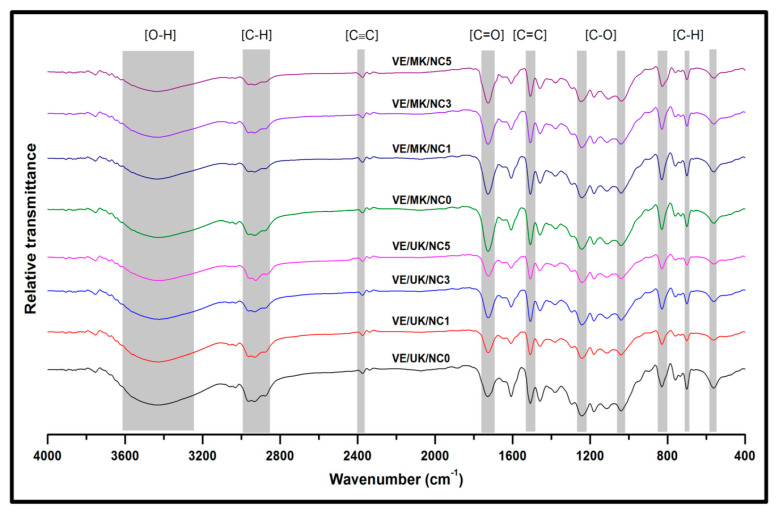
FT-IR analysis of modified and unmodified kenaf fibre enhanced nanocomposites.

The chemical composition of the unmodified kenaf reinforced nanocomposite and modified kenaf reinforced nanocomposite was indicated using functional groups from the FT-IR analysis in Figure 10. The bands around 3200 and 3500 cm^−1^ are assigned to O–H stretching vibrations. A slight increase of C=O and C=C stretching vibration around 1650 and 1550 cm^−1^ was achieved for the modified kenaf fibre-based bionanocomposite compared to the unmodified kenaf bionanocomposite, respectively. The increase in peaks of the stretching band attributed to C=O vibrations indicated increased interaction between the hydroxyl groups in the kenaf fibres and the effect of the propionic anyhdride modification [51]. The band which corresponds to C–H asymmetric stretching vibration occurred at 2900–2950 cm^−1^. The bands around 820 cm^−1^ corresponds to CH out-of-plane bending vibrations in substituted ethylenic systems. 

#### 3.3.5. Thermal Properties

**Figure 11:**
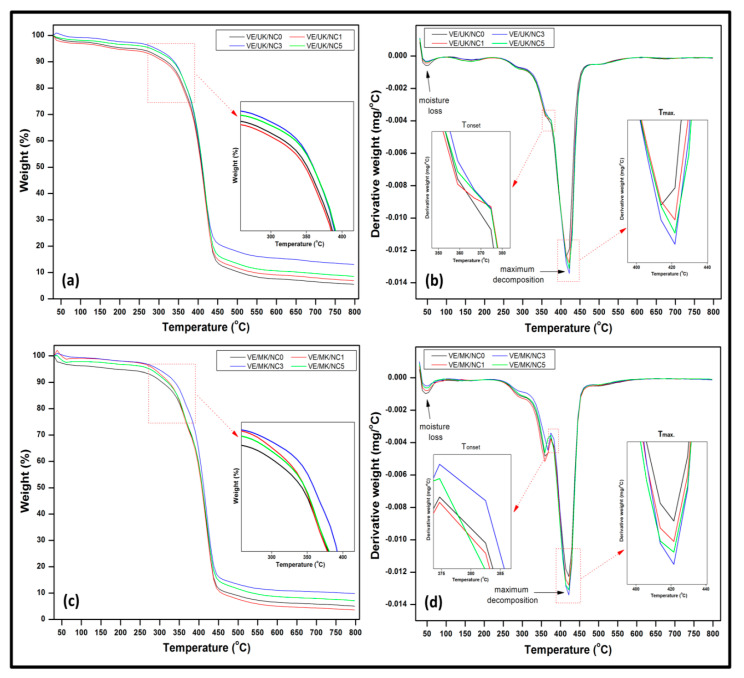
Thermal properties of (**a**) TGA profile of unmodified nanocomposite (**b**) DTG profile of unmodified nanocomposite (**c**) TGA profile of modified nanocomposite (**d**) DTG profile of modified nanocomposite.

The thermal properties of the unmodified and modified kenaf-based composite were analysed from the temperature profile. The result was presented in terms of weight loss and derivative weight loss (DTG) in Figure 11. It is indicated from the initial weight loss from the TG curve that evaporation occurred around 50 to 100 °C for all samples indicating that dehydration of the samples is likely to have occurred. The second stage of thermal degradation occurred in the range of 400 to 500 °C for all samples, at this stage, maximum weight loss was achieved due to the effect of volatilisation of filler (kenaf fibre) in the matrix. This stage was characterised by the decomposition of the hemicellulose, cellulose and lignin which are the three major constituent of kenaf in the nanocomposite structure [52]. It was revealed that the weight loss increased as the filler loading was increased in the unmodified nanocomposite (Figure 11a) which implies that the unmodified nanocomposite exhibited increased rate of degradation. As indicated in the DTG curve in Figure 11b, increase in filler loading at 3% exhibited higher decomposition temperature from the Tonset and Tmax temperature profile. Further increase in the filler loading resulted in decrease in the decomposition of the fibres in the unmodified nanocomposite. However, the rate of degradation was lower in terms of the weight loss as illustrated in the modified nanocomposite compared to unmodified nanocomposite (Figure 11c). As the filler loading increased, weight loss increased although it was found lower compared to the unmodified nanocomposite. The initial degradation temperature (Tonset) for composites of unmodified fibres shifted to a higher temperature, compared to the modified fibre indicating higher thermal stabilities of the modified fibres. This result suggested that propionylated composites consisted of well embedded structure as filler loading increased and could form a good bonding interfacial interaction between the fibre and matrix. Finally, as the temperature was increased from 500 to 800 °C, dehydroxylation of bio nanocarbon occurred. It was indicated from the plot that the addition of activated bio-nanocarbon provided thermal stability to the composite due to the effect of its dispersion in the vinyl–kenaf composite structure which resulted in a decrease in weight loss compared to unmodified vinyl ester–kenaf composites, irrespective of the nanofiller loading. 

A summary from the numerical data obtained from the thermogravimetric temperature profile is illustrated in the Table 2. It was indicated that the temperature of decomposition (Tonset) and maximum temperature of decomposition (Tmax) exhibited noticeable shift to higher temperature as the filler loading increased. It was revealed that increase in filler loading resulted in increase in the decomposition temperature until 3% after which decrease in the Tonset and Tmax was achieved at equivalent 376.78 and 420.74 °C. It was indicated that percent mass loss decreased as the filler loading increased. In general, the nanocomposite exhibited optimum decomposition temperature at 3% filler loading with decrease in percent mass loss at the temperature increased from 100 to 800 °C. 

## 4. Conclusions

Propionic anhydride was used as modification agent of kenaf fibre in vinyl ester matrix to form bionanocomposites and was compared to unmodified kenaf fibre-reinforced nanocomposite. The bionanocarbon used as nanofiller in the fibre–matrix networks varied from 0 to 5% in an increment of 2% by weight in the composites. The surface morphology, wettability, structural, functional, tensile and thermal properties were investigated using the FESEM, TEM, Contact angle, Density profile, FT-IR and TGA. Improved functional properties of the modified bionanocomposite were achieved compared to the unmodified bionanocomposite. The water absorption and thickness swelling decreased as the filler loading increased relative to the control sample. The decrease was attributed to the addition of nanofiller in the fibre–matrix interaction within the nanocomposite networks. It was indicated that the structural, functional and thermal properties of the modified kenaf-derived bionanocomposite were better improved compared to unmodified kenaf reinforced bionanocomposite at 3% filler loading. This was attributed to better compatibility of fibre–matrix interaction, as indicated in the morphological features. The effect of propionic anhydride modification reduced the impurities and non-polar compounds of the fibre, resulting in better interfacial interaction between the fibre matrix. The incorporation of bionanocarbon across the fibre–matrix interface filled the voids and hence improved the interfacial interaction in the composite. Hence, enhanced compatibility between the fibre–matrix interactions and improved the functional properties of the bionanocomposites was achieved. 

## Figures and Tables

**Table 1:** Mechanical properties of unmodified and modified kenaf reinforced nanocomposite.

Mechanical Properties	VE/UK/NC0	VE/UK/NC1	VE/UK/NC3	VE/UK/NC5	VE/MK/NC0	VE/MK/NC1	VE/MK/NC3	VE/MK/NC5
Tensile strength (MPa)	47.96 ± 1.08	54.79 ± 1.26	68.65 ± 0.79	62.75 ± 0.74	57.50 ± 1.04	65.36 ± 0.77	78.61 ± 1.02	73.09 ± 1.28
Tensile modulus (GPa)	1.75 ± 0.09	2.17 ± 0.08	2.65 ± 0.06	2.46 ± 0.04	2.24 ± 0.12	2.56 ± 0.08	2.87 ± 0.07	2.77 ± 0.06
Elongation at break (%)	7.23 ± 0.38	7.08 ± 0.24	5.62 ± 0.32	5.93 ± 0.14	6.36 ± 0.22	5.77 ± 0.13	5.38 ± 0.21	5.45 ± 0.18
Tensile toughness (MPa)	80.31 ± 1.95	83.75 ± 1.43	92.67 ± 0.61	88.18 ± 1.03	86.28 ± 1.05	90.63 ± 0.97	95.37 ± 1.59	93.72 ± 0.60
Flexural strength (MPa)	64.71 ± 1.12	68.04 ± 1.05	88.23 ± 1.31	81.60 ± 0.94	78.52 ± 1.52	84.74 ± 1.78	96.81 ± 1.27	91.10 ± 1.32
Flexural modulus (GPa)	4.68 ± 0.17	5.86 ± 0.09	7.53 ± 0.16	6.35 ± 0.08	6.26 ± 0.04	7.32 ± 0.13	8.38 ± 0.14	8.02 ± 0.16
Flexural toughness (GPa)	62.41 ± 1.50	64.71 ± 1.25	78.79 ± 0.81	72.05 ± 0.58	68.75 ± 1.22	74.78 ± 0.78	85.68 ± 0.93	81.07 ± 1.22
Impact strength (kJ/m^2^)	4.36 ± 0.19	4.73 ± 0.15	5.89 ± 0.08	5.46 ± 0.15	5.05 ± 0.15	5.46 ± 0.12	6.75 ± 0.18	6.27 ± 0.19

**Table 2:** Decomposition temperatures and mass loss data of nanocomposite.

Composites	Decomposition Temperature (°C)		Mass Loss (%)			
	T_onset_	T_max._	100 °C	375 °C	500 °C	800 °C
VE/UK/NC0	372.64	418.36	3.09	28.39	92.28	96.40
VE/UK/NC1	374.20	420.18	3.75	28.15	90.98	94.98
VE/UK/NC3	380.51	421.56	0.98	25.09	86.58	91.44
VE/UK/NC5	376.78	420.74	1.81	27.56	88.25	93.09
Average	376.03	420.21	2.41	27.30	89.52	93.98
Difference			22	4	3	3
VE/MK/NC0	392.95	425.94	2.45	23.99	88.38	92.85
VE/MK/NC1	396.59	426.93	0.79	21.47	89.65	94.48
VE/MK/NC3	408.74	428.89	0.68	20.23	81.99	86.96
VE/MK/NC5	405.36	427.75	2.16	21.30	86.64	90.15
Average	400.91	427.38	1.52	21.75	86.67	91.11
Difference			38	9	2	2

## Data Availability

Not applicable.

## Sample Availability

Samples of the compounds are available from the authors.
